# Supplementary material for: Assessing target genes for homing suppression gene drive
Source: EMBO J. 2026 Feb 6;45(6):2074–94. doi: 10.1038/s44318-025-00683-y (PMC12992549; doi:10.1038/s44318-025-00683-y)
Supplement: Supplementary file 1 — Table EV1 [file 44318_2025_683_MOESM1_ESM.docx]

**Table EV1 Target gene information**

| **Target gene** | **Gene ID** | **Chromosome** | **Function** |
| --- | --- | --- | --- |
| *ix* (*intersex*) | CG13201 | 2R | Female fertility/viability; Required for female sexual development |
|  |  |  |  |
|  |  |  |  |
|  |  |  |  |
| *ndl* (*nudel*) | CG10129 | 3L | Female viability/maternal effect; Required for both eggshell biogenesis and embryonic dorsoventral patterning |
|  |  |  |  |
|  |  |  |  |
|  |  |  |  |
| *nox* (*NADPH oxidase*) | CG34399 | 2R | Female fertility; Involved in calcium flux and smooth muscle contraction during ovulation |
|  |  |  |  |
|  |  |  |  |
|  |  |  |  |
| *oct* (*Octopamine β2 receptor*) | CG33976 | 3R | Female fertility; Essential for ovulation and fertilization |
|  |  |  |  |
|  |  |  |  |
|  |  |  |  |
| *stl* (*stall*) | CG3622 | 2R | Female fertility; Involved in neuron migration inhibition and ovarian follicle development. |
|  |  |  |  |
|  |  |  |  |
|  |  |  |  |
| *tra* (*transformer*) | CG16724 | 3L | Female fertility; Controls female sexual differentiation |
|  |  |  |  |
|  |  |  |  |
|  |  |  |  |
|  |  |  |  |
|  |  |  |  |
| *vir* (*virilizer*) | CG3496 | 2R | Female fertility/viability; Required for sex determination and dosage compensation via *sxl* alternative splicing |
|  |  |  |  |
|  |  |  |  |
|  |  |  |  |
| *dec* (*defective chorion*) | CG2175 | X | Female fertility; Required for proper assembly of the eggshell |
|  |  |  |  |
|  |  |  |  |
|  |  |  |  |
| *sxl* (*sex-lethal*) | CG43770 | X | Female fertility/viability; Required for sex determination |
|  |  |  |  |
|  |  |  |  |
|  |  |  |  |
