# Supplementary material for: Assessing target genes for homing suppression gene drive
Source: EMBO J. 2026 Feb 6;45(6):2074–94. doi: 10.1038/s44318-025-00683-y (PMC12992549; doi:10.1038/s44318-025-00683-y)
Supplement: Supplementary file 2 — Table EV2 [file 44318_2025_683_MOESM2_ESM.docx]

**Table EV2 Homologs of target genes across different insect species.**

| Species name | Protein access number of target gene | | | | | | | |
| --- | --- | --- | --- | --- | --- | --- | --- | --- |
|  | *dec* | *ix* | *nox* | *oct* | *stl* | *sxl* | *tra* | *vir* |
| *Drosophila melanogaster* | NP 727202.1 | NP_610677.1 | AEO20323.1 | NP_001163596.2 | NP_001097423.1 | NP_001259303.1 | NP 524114.1 | NP_524900.1 |
| *Ceratitis capitata* | XP 020715373.1 | XP_004537514.1 | XP_020717669.1 | XP_020713689.1 | XP_012154838.1 | XP_012158509.1 | XP 020714997.1 | XP_012157537.1 |
| *Spodoptera frugiperda* | - | XP_035453121.1 | XP_035454891.2 | XP_035441849.1 | XP_050551061.1 | XP_035448223.1 | - | XP_050549381.1 |
| *Plutella xylostella* | - | XP_037962558.1 | XP_048487167.1 | XP_037967636.1 | XP_048483858.1 | XP_048482129.1 | - | WQH20077.1 |
| *Aedes aegypti* | - | XP_001654364.1 | XP_001660738.2 | XP_021693342.1 | XP_001660627.2 | XP_021704539.1 | - | XP_001660468.2 |
| *Anopheles gambiae* | - | XP_321918.4 | XP_061517613.1 | XP_003436280.1 | XP_061505633.1 | XP_061505793.1 | - | XP_061514589.1 |
| *Culex quinquefasciatus* | - | XP_001849607.1 | XP_038111558.1 | XP_038115039.1 | XP_038120942.1 | XP_038120092.1 | - | XP_038111858.1 |
| *Leptinotarsa decemlineata* | - | XP_023028483.1 | XP_074037292.1 | XP_074032976.1 | XP_074032509.1 | XP_023012638.1 | - | XP_023012377.2 |
| *Rhynchophorus ferrugineus* | - | KAF7271975.1 | KAF7267617.1 | KAF7274530.1 | KAF7280146.1 | KAF7286598.1 | - | KAF7273791.1 |
| *Periplaneta americana* | - | XP_069676739.1 | XP_069681706.1 | XP_069695476.1 | XP_069681719.1 | XP_069677446.1 | KAJ4447572.1 | XP_069688161.1 |
| *Blattella germanica* | - | PSN42758.1 | PSN47090.1 | PSN51957.1 | PSN53910.1 | PSN30343.1 | QGB21093.1 | PSN36139.1 |

“-” indicates no hit.
