# Supplementary material for: Assessing target genes for homing suppression gene drive
Source: EMBO J. 2026 Feb 6;45(6):2074–94. doi: 10.1038/s44318-025-00683-y (PMC12992549; doi:10.1038/s44318-025-00683-y)
Supplement: Supplementary file 3 — Table EV3 [file 44318_2025_683_MOESM3_ESM.docx]

**Table EV3 Sequencing of drive females in embryo resistance assessment**

| **Target gene** | **gRNA1** | **gRNA2** | **gRNA3** | **gRNA4** |
| --- | --- | --- | --- | --- |
| Nox | WT | WT | mosaic | mosaic |
|  | R | - | - | R |
|  | mosaic | WT | mosaic | mosaic |
|  | WT | WT | mosaic | mosaic |
|  | WT | WT | WT | mosaic |
|  | R | WT | WT | WT |
| Dec | R | WT | WT | mosaic |
|  | mosaic | mosaic | WT | WT |
|  | mosaic | mosaic | WT | WT |
|  | R | WT | WT | WT |
|  | R | WT | WT | WT |
|  | mosaic | WT | WT | WT |
|  | R | WT | WT | R |
|  | R | WT | WT | WT |
|  | R | WT | WT | WT |
|  | R | WT | WT | WT |
|  | R | mosaic | mosaic | WT |
|  | mosaic | WT | WT | WT |
| Oct | WT | WT | mosaic | mosaic |
|  | mosaic | R | mosaic | - |
|  | WT | WT | mosaic | WT |
|  | R | - | R | mosaic |
|  | WT | WT | WT | WT |
|  | WT | WT | WT | WT |
|  | WT | WT | WT | WT |
|  | R | R | - | - |
|  | WT | WT | WT | WT |
|  | R | mosaic | mosaic | WT |
|  | WT | WT | WT | WT |
| Stl | - | - | - | R |
|  | R | WT | mosaic | mosaic |
|  | R | - | - | WT |
|  | R | WT | R | WT |
|  | mosaic | WT | R | WT |
|  | WT | WT | WT | WT |
|  | mosaic | WT | mosaic | WT |
|  | mosaic | WT | mosaic | mosaic |
|  | mosaic | WT | mosaic | mosaic |
|  | WT | WT | R | mosaic |
|  | mosaic | WT | mosaic | mosaic |
|  | mosaic | WT | R | mosaic |
|  | R | - | - | mosaic |
| stl.f1(fertile) | WT | WT | mosaic | WT |
| stl.f3(fertile) | WT | WT | mosaic | WT |
| stl.f4(fertile) | WT | WT | WT | WT |
| Tra-v1 | WT | mosaic | mosaic | WT |
|  | WT | mosaic | mosaic | WT |
|  | WT | mosaic | mosaic | WT |
|  | WT | mosaic | R | WT |
|  | WT | R- | mosaic | WT |
|  | WT | mosaic | - | WT |
|  | WT | mosaic | mosaic | WT |
|  | WT | mosaic | mosaic | WT |
|  | WT | R- | R | WT |
|  | WT | mosaic | mosaic | WT |
|  | WT | mosaic | mosaic | WT |
|  | mosaic | mosaic | mosaic | WT |
|  | WT | mosaic | mosaic | WT |
|  | WT | mosaic | WT | WT |
|  | WT | mosaic | mosaic | WT |
|  | mosaic | mosaic | mosaic | WT |

*Tested females were all sterile unless specified, and the sequencing was conducted with both forward and reverse primers. ‘WT’ indicates wildtype allele and ‘mosaic’ means the existence of multiple mutant alleles, while ‘R’ and ‘-’ respectively indicate resistance (indel) and deletion covering the whole target site.
