# Supplementary material for: Assessing target genes for homing suppression gene drive
Source: EMBO J. 2026 Feb 6;45(6):2074–94. doi: 10.1038/s44318-025-00683-y (PMC12992549; doi:10.1038/s44318-025-00683-y)
Supplement: Supplementary file 4 — Table EV4 [file 44318_2025_683_MOESM4_ESM.docx]

**Table EV4 Maximum-likelihood analysis of fitness in cage populations**

| **Cage** | **% Effective population size** | | | **Female heterozygote fitness** | | |
| --- | --- | --- | --- | --- | --- | --- |
| ***nox*/*nanos*** | 0.030 | [0.005 | 0.084] | 0.262 | [0.000 | 0.664] |
| ***oct*/*nanos*** | 0.025 | [0.007 | 0.057] | 0.245 | [0.000 | 0.664] |
| ***oct*/*CG4415*** | 0.046 | [0.021 | 0.085] | 0.318 | [0.166 | 0.508] |
| **s*tl*/*nanos-*cage1** | 0.160 | [0.032 | 0.421] | 0.681 | [0.426 | 1.065] |
| ***stl*/*nanos-*cage2** | 0.261 | [0.072 | 0.638] | 0.205 | [0.098 | 0.327] |

Brackets indicate 95% confidence interval.
