# Supplementary material for: Assessing target genes for homing suppression gene drive
Source: EMBO J. 2026 Feb 6;45(6):2074–94. doi: 10.1038/s44318-025-00683-y (PMC12992549; doi:10.1038/s44318-025-00683-y)
Supplement: Supplementary file 13 — Expanded View Figures [file 44318_2025_683_MOESM13_ESM.pdf]

Expanded View Figures

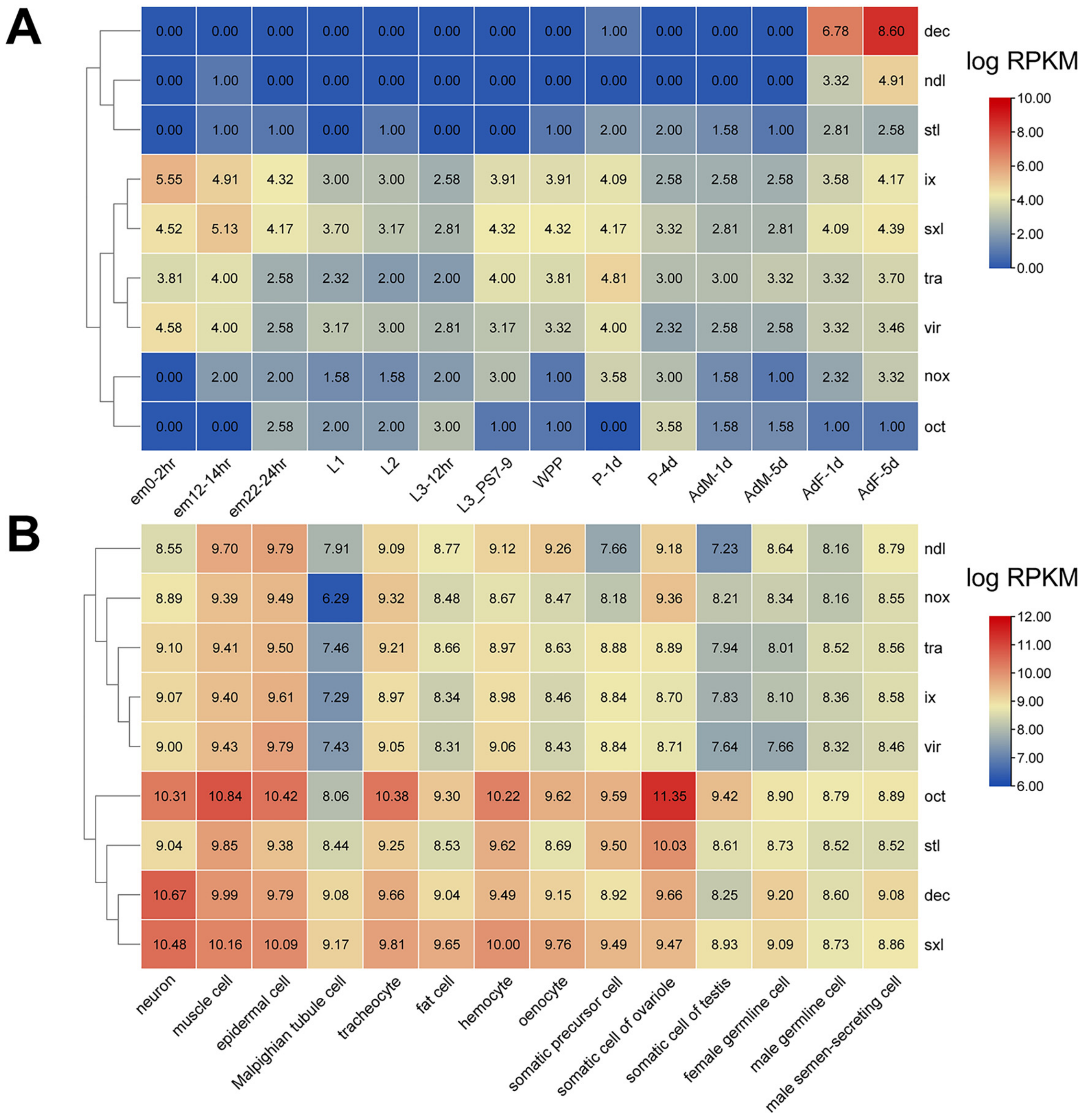

Figure EV1. Expression profile of target genes.

(**A**) Expression pattern in different developmental stages. em embryo, L larva, WPP white prepupa, P pupa, AdM adult male, AdF adult female. (**B**) Expression pattern in different adult cell types. The values inside the heatmap indicate expression levels of target genes (log2(RPKM + 1)). Red color indicates high expression, and blue color represents low expression. Genes with similar expression profiles are clustered together.

© The Author(s)

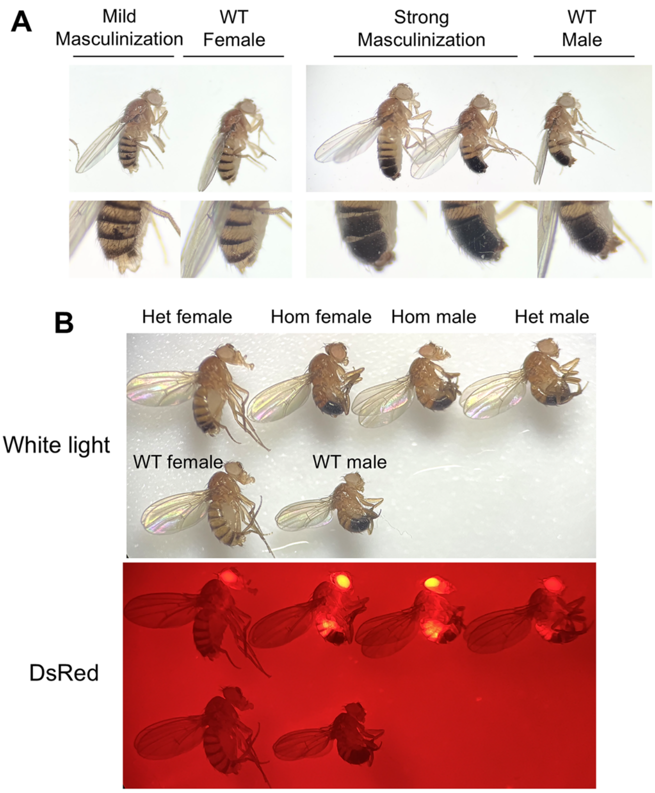

Figure EV2. Phenotypes of drive targeting *tra*.

(**A**) After crossing flies heterozygous for both *tra*-targeting drive and Cas9 alleles with w^{1118}, two abnormal phenotypes were observed in their offspring. These were genetically female but exhibited various levels of masculinization. We defined unusual patchy pigmentation in dorsal fragments as mild masculinization, while male-like flies were defined as strong masculinization. (**B**) After intercrossing drive heterozygotes (Het) in the absence of Cas9, homozygous (Hom) females showed male phenotype, while heterozygotes were identical to w^{1118} flies (wild-type, WT). Homozygosity is indicated by strong fluorescence and confirmed by genotyping.

 © The Author(s)

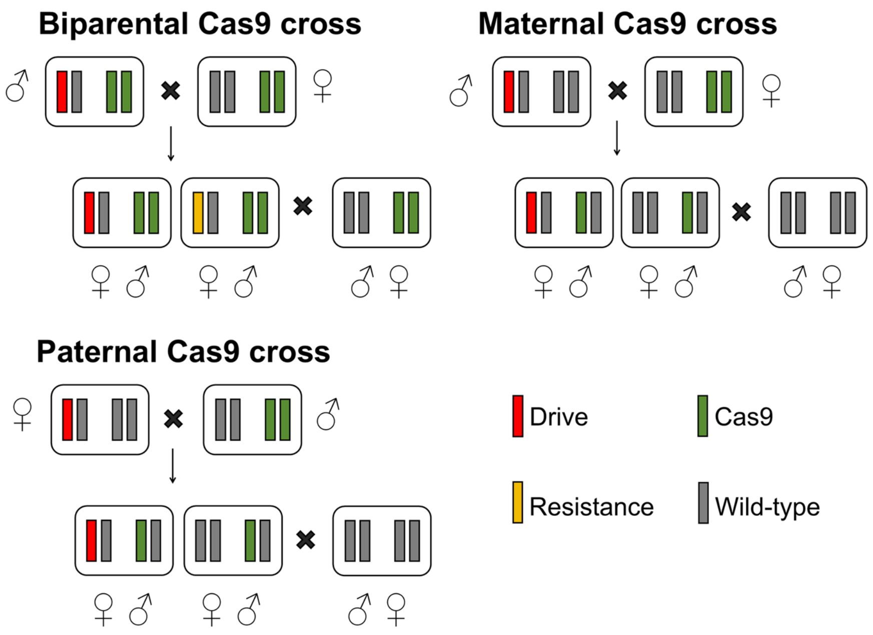

Figure EV3. Illustration of cross schemes for fecundity and fertility tests.

The biparental Cas9 cross was specifically designed for assessing drive fitness in cage populations. In the initial generation, males heterozygous for drive and homozygous for Cas9 were crossed to Cas9 homozygous females to generate drive and non-drive flies for fecundity and fertility assessment. In this case, the fitness of drive females was likely reduced by maternally deposited Cas9 and zygotically expressed gRNA. In maternal and paternal Cas9 crosses, Cas9 was provided by female and male flies, respectively. Biparental and maternal crosses have identical maternal effects in their female offspring, which are not present in the parental cross. Fitness costs from somatic Cas9 expression would still be present in all crosses, and these are likely higher in the biparental cross.

© The Author(s)

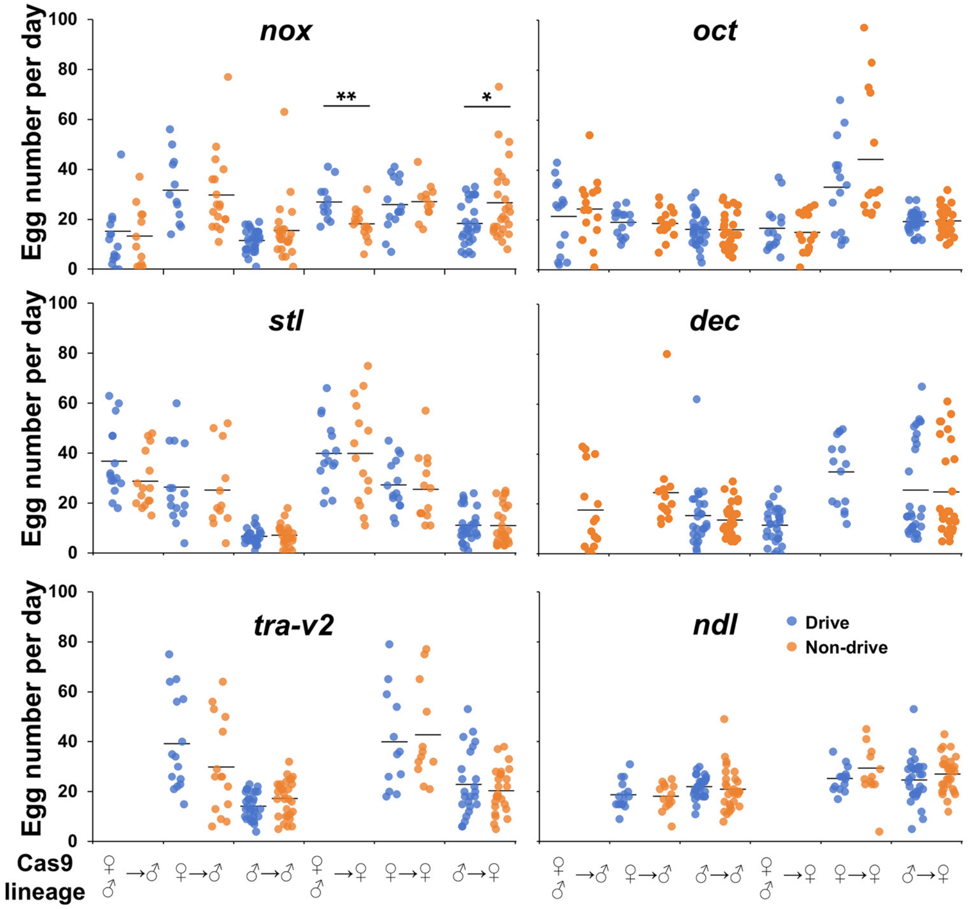

Figure EV4. Female fecundity.

Three cross schemes were applied, as shown by Cas9 lineage. For biparental Cas9, crosses were made between male drive heterozygotes and wild-type females at the drive site, and both parents were homozygous for Cas9. For maternal or paternal Cas9 crosses, either drive heterozygote males or females (without Cas9) were crossed to opposite sex flies that were homozygous for Cas9. For all three crosses, drive and non-drive progeny were then crossed to non-drive flies, and the offspring were phenotyped. Drive and non-drive flies are marked with dots in different colors. Biparental Cas9 cross data was not collected for *tra-v2* and *ndl*, and only partially collected for X-linked *dec*. Drive and non-drive flies are marked with dots in different colors. Significant difference is indicated with **p* < 0.05, ***p* < 0.01 (*t*-test). Raw data is provided in Datasets EV4, EV5, and EV6.

© The Author(s)

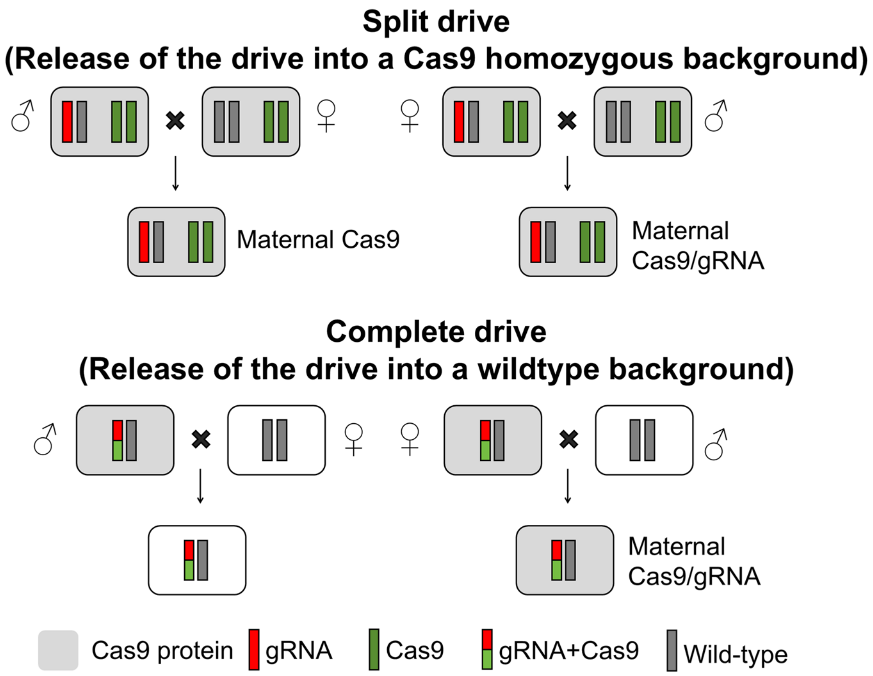

Figure EV5. Comparison between split drive and complete mechanisms.

In this study, flies carrying the drive element (i.e., the gRNA) were introduced into a population that is homozygous for Cas9 (upper half), representing a similar complete drive (lower half). However, there are potentially important differences. First, two copies of Cas9 are provided in all individuals for the split drive, and they are at a different genomic site unlinked to gRNA. Second, offspring will always receive maternal Cas9, which is not the case for the complete drive. This maternal Cas9 can combine with newly expressed gRNA, producing resistance allele formation or drive conversion in somatic cells. The lack of wild-type alleles in such cells can have negative fitness effects on females.

© The Author(s)
